# Evaluation of left ventricular systolic function in children with sickle cell anemia: contribution of 2D strain

**DOI:** 10.12688/f1000research.125345.1

**Published:** 2022-10-24

**Authors:** Sarra Chenik, Aymen Noamen, Abyr Bouslimi, Houaida Mahfoudhi, Sadok Hannachi, Hager Barakizou, Islam Mejri, Tasnim Znegui, Wafa Fehri

**Affiliations:** 1 Cardiology department, Military Hospital of Tunis, Tunis, Tunisia; 2 Cardiology department, Nantes Hospital,France,, Nantes, France; 3 Pediatric department, Military Hospital of Tunis, Tunis, Tunisia; 4 Pneumology department, Military Hospital of Tunis, Tunis, Tunisia

**Keywords:** sickle cell anemia; heart disease; echocardiography; Speckle tracking echocardiography. Global longitudinal strain; Left ventricular systolic function; child.

## Abstract

Background:

Cardiovascular involvement is not well studied in children with sickle cell disease. The aim of this study was to evaluate the echocardiographic parameters and to investigate speckle tracking echocardiography (STE) interest in detecting subclinical myocardial impairment of children with sickle cell disease.

Methods:

The study was directed in the echocardiographic laboratory in the military hospital of Tunis between July 2018 and December 2018. 30 patients with sickle cell anemia (SCA) and 30 controls were compared. The echocardiographic measurements were indexed according to body surface. Cardiac output, left ventricular ejection fraction, wall thickness, as well as LV 2-D longitudinal systolic strain were assessed.

Results:

The SCA Group included 30 patients (11.8 ± 2yrs, sex ratio: 1.31) with homozygous SCA and the C Group included 30 healthy controls (12.7 ± 1,2yrs, sex ratio: 1.27).

According to the findings, SCA Group showed significantly larger LV diameter (36.2±2.5mm/m2 vs 29.3±1.3mm/m2, p=0.005). SCA Group also showed lower LV ejection fraction (62%±0.5 vs 65%±5, p=0.001). No significant difference was observed for cardiac output (p=0.4). Otherwise, two-dimensional longitudinal strain of LV was higher in SCA group (-21%±3.07 vs -25%±2.98; p<0.01).

Conclusions:

Our study highlights several cardiac abnormalities in children with SCA, which could represent a marker of disease severity and point out the importance of the cardiologic screening of these patients.

## List of abreviations

2D: two-dimensional

C: Control

DTI: Pulsed Doppler tissue

GLS: global longitudinal strain

IVST: interventricular septal wall thickness

LV: Left ventricular

LVEDD: Left ventricular end-diastolic diameter

LVEF: Left ventricular ejection fraction

LVESD: Left ventricular end-systolic diameter

LVGLS: Left ventricular global longitudinal strain

LVM: Left ventricular mass

MAPSE: mitral annular plane systolic excursion

PWT: Posterior wall thickness

S’: systolic mitral annulus velocity

SCA: Sickle cell anemia

TM: Time Movement

TTE: transthoracic echocardiography

## Introduction

Sickle cell disease or sickle cell anemia is an autosomal recessive genetic disease linked to a hemoglobin abnormality leading to the deformation of red blood cells.

The disease affects more than 50 million people worldwide, particularly in black Africa and the Mediterranean region. ^1^ Globally, hemoglobin disorders are responsible for about 3.4% of deaths in children under 5 years of age. ^1^^,^^2^

Nevertheless, the life expectancy of sickle cell patients has improved in recent years with healthier lifestyles, medical monitoring, and new therapies. The increase in their life expectancy has led to the appearance of chronic multi-systemic complications in addition to acute complications. Indeed, despite a common genetic basis and a similar physiopathology, sickle cell patients have a clinical phenotype of very variable severity.

It is therefore a serious chronic disease whose prognosis depends on the occurrence of complications, particularly cardiovascular ones. However, the discovery of this cardiovascular disorder is often made in adulthood, and the literature is poor on its detection in childhood, probably because of the lack of systematic cardiological follow-up in these children.

Nowadays, with the advent of new echocardiographic techniques such as two-dimensional (2D) strain, it has been possible to demonstrate systolic and diastolic dysfunction earlier than with conventional echocardiography.

The advent of 2D strain has enabled early detection of cardiac damage in many previously unrecognized chronic conditions and is increasingly a prognostic marker to guide patient management.

It is a new technique that studies myocardial motion by tracking speckles, which are acoustic markers of the myocardium. Local tissue motion is represented by the geometric displacement of each speckle. Several software packages have been developed to allow temporal and spatial processing of the image obtained by the 2D strain.

Thus, we undertook this study of sonographic aspects of the hearts of children with sickle cell disease, to identify the different possible abnormalities detected in the left ventricular function by conventional ultrasound and the 2D strain technique in order to assess cardiac injury and establish a clinical and echocardiographic monitoring scheme.

The main objective of our study is to evaluate the interest of 2D strain in the detection of left ventricular (LV) myocardial damage at a sub-clinical stage in children with sickle cell disease.

## Methods

### Study subjects and design

This is a descriptive cross-sectional study conducted in the cardiology department at the Main Military Hospital of Instructions in Tunis which took place over six months between July 2018 and December 2018.

### Study population

*Sample size calculation*

We estimated that 2.4% of patients were followed up at the pediatric department and referred to pediatric cardiology consultation during the study period. A sample size of 35 patients would be required to achieve statistical significance (power: 0.8; alpha: 0.05) calculated using a predictive formula. ^3^

*Inclusion criteria of the SCA group*

We included first the SCA group comprising 30 children followed up at the pediatric department of the Tunis military hospital and who benefited from transthoracic echocardiography (TTE) during their follow-up.

*Non-inclusion criteria of the SCA group*

We did not include patients with congenital heart disease, associated valvular heart disease, or those who had undergone cardiac surgery (n=1). We also did not include two patients who were lost to follow-up at the time of the study and one participant who requested to withdraw from the study.

*Exclusion criteria*

Excluded from this study were patients with poor echogenicity during TTE (n-=).

*Control group*

Our study included a second group “C” of 30 controls free of any cardiovascular or respiratory pathology and not presenting anemia at the time of the study.

These were children who had been hospitalized in the pediatric department and referred to a pediatric cardiology consultation for the assessment of a heart murmur or for the exploration of atypical chest pain, and who had a normal echocardiography.

The control group was then matched according to age, gender and body mass index with the SCA group.

### Data collection

The participants were contacted by phone and were identified by name and date of birth, and then anonymized during data entry. Data collection was carried out by a single physician using a patient information sheet. All parents seemed to find participation in the study beneficial.

They agreed to participate, and their children underwent an echocardiographic examination because they found it non-invasive and important for disease assessment.

*Clinical and paraclinical data*

Data collected were age, gender, and medical history. A study of the medical records of sickle cell patients was carried out and allowed us to collect clinical and paraclinical data such as: age of discovery of the disease, baseline hemoglobin level, rate of transfusion, existence of complications and treatments received.

*Echocardiography*

All study subjects from both SCA and control groups underwent echocardiographic examination and speckle tracking measurement, all performed by a single experienced cardiologist who was blinded to all clinical data.

This examination was performed using a vivid E7 ultrasound system (GE healthcare, Horten, Norway) with an S 5-1 probe according to the guidelines of the American Society of Echocardiography and the European Association of Cardiovascular Imaging (12). Measurements were performed in the parasternal long axis, parasternal short axis, apical two, three, four, five cavities and subcostal. During the examination the scale was set between 12 and 20 cm/s to avoid spectral folding.

Each incidence was taken so that the angle between the myocardial wall under study and the ultrasound beam does not exceed 30 degrees. All echographic data was then stored on a central memory unit, allowing for the post-processing and adjustment of measurements, including measurement work on pulsed Doppler tissue (DTI) spectra by averaging the results of three to five cardiac cycles.

Left ventricular study

Left ventricular end-diastolic diameter (LVEDD), left ventricular end-systolic diameter (LVESD), interventricular septal wall thickness (IVST) and posterior wall thickness (PWT) were measured with 2D targeted M-mode tracing. Left ventricular mass (LVM) was estimated using Devereux formula.

Left ventricular ejection fraction (LVEF) was calculated using Simpson’s biplane method of discs then systolic mitral annulus velocity (S’) was assessed using tissue Doppler imaging in 2D mode of the mitral annulus in the four-chamber view.

Strain analysis by speckle-tracking echocardiography: myocardial deformation study

Speckle tracking analysis was conducted offline on the apical four chamber, apical long axis, and apical two chamber views that were previously stored in Digital Imaging and Communications in Medicine (DICOM) format. Analysis of echocardiographic images was performed offline using Echo PAC (GE Medical Systems, Norway).

Three cardiac cycles were recorded in cine loop format at a frame rate between 50 and 80 frames per second as two-dimensional grayscale views.

One end systolic frame is selected by the operator to perform the manual tracing of the endocardial border of the left ventricle (LV), this tracing is then used by the software to create a region of interest. The myocardium is then automatically divided in segments according to the standard 16-segment model of the LV. ^4^

**Figure 1.**
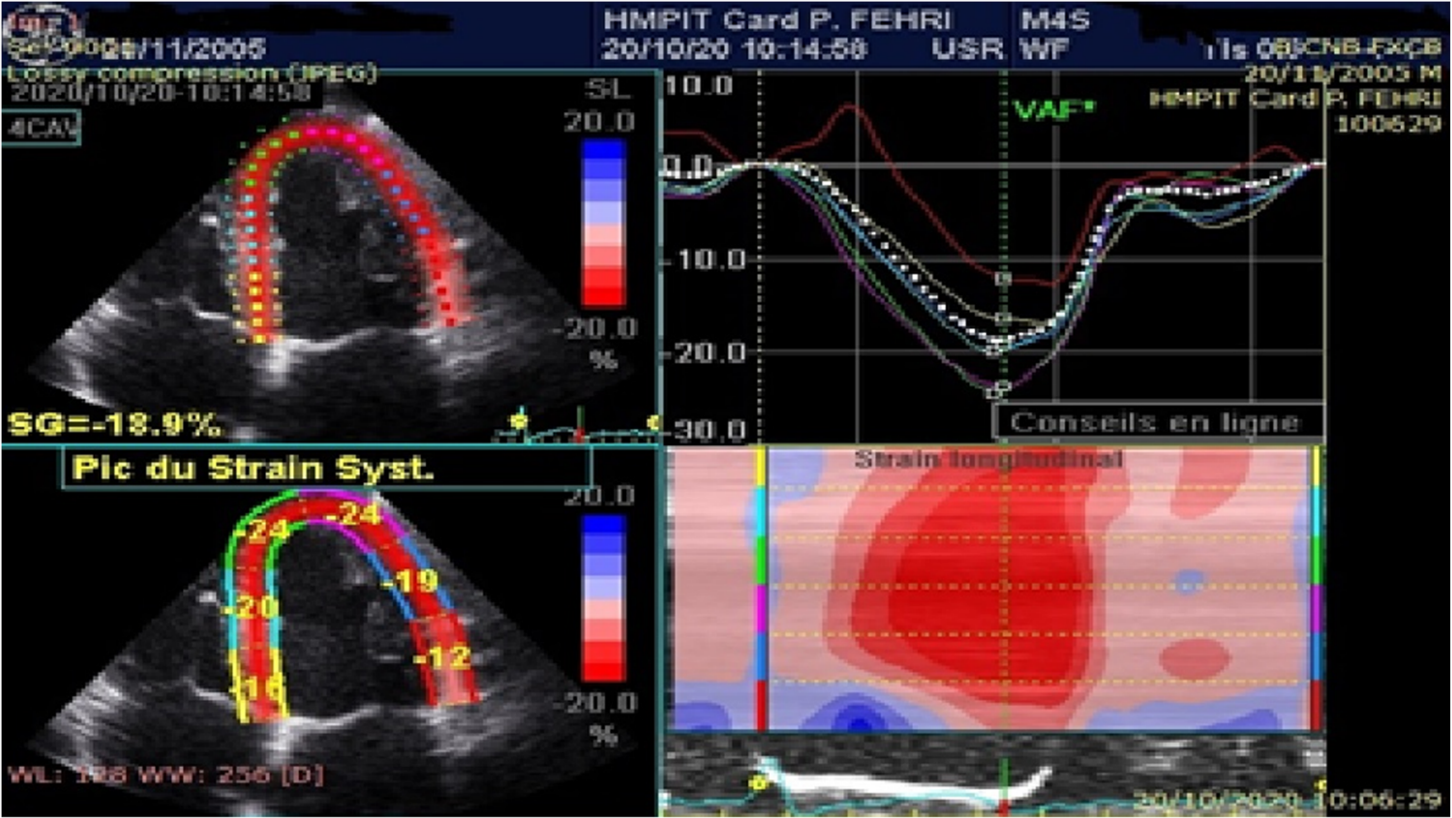
Analysis of longitudinal deformations from the apical incidence of 4 cavities in a healthy subject.

**Figure 2.**
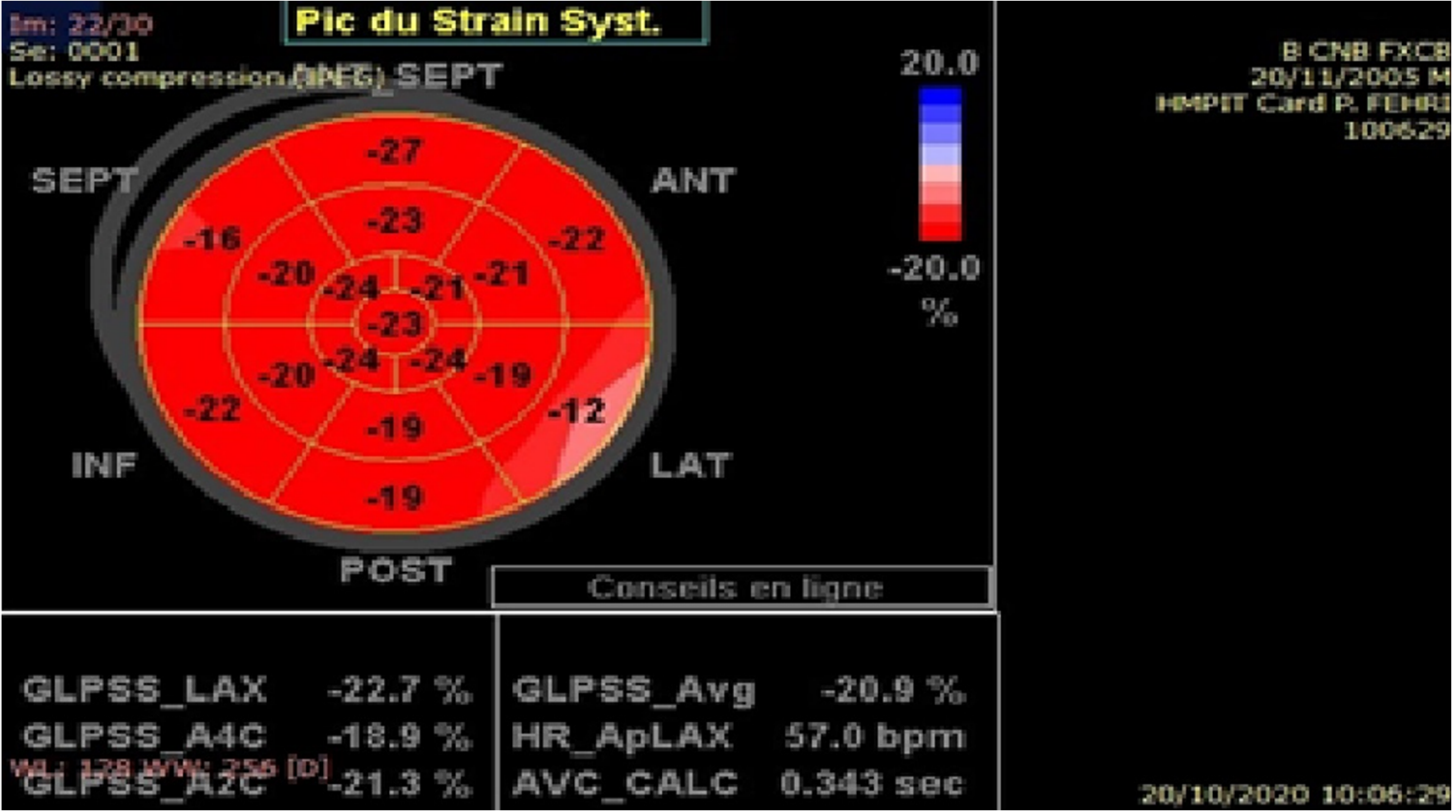
Segmental and global left ventricular longitudinal strain in a healthy subject.

The quality of myocardial tracking was visually checked in real time then manually corrected to ensure optimal tracking. The software then tracks the deformation of the myocardium during cardiac cycle to calculate peak systolic segmental strain. Global longitudinal strain (GLS) is determined as the average of the segmental strains ( Figures 1, 2).

### Statistical analysis

All statistical analyses were conducted using SPSS 25.0 software (IBM SPSS Inc., Chicago, Illinois, USA). Quantitative variables were tested for normal distribution using the Shapiro-Wilk and Kolmogorov-Smirnov tests then expressed as mean±standard deviation. Qualitative variables were expressed as counts and percentages.

Parametric data was then compared using Student’s T test for independent-samples and non-parametric data was compared using the Mann-Whitney-Wilcoxon test.

Potential relation between clinical or echocardiographic characteristics of SCA patients on GLS measurement was assessed using chi-square or Fisher’s exact test for qualitative data and Pearson’s correlation coefficient and Spearman’s rank correlation coefficient for quantitative data. All statistical tests were two-sided, and the level of statistical significance was set up at p<0.05.

### Literature review

Bibliographic research was undertaken on PubMed through MESH research using keywords: sickle cell anemia; heart disease; echocardiography; Speckle tracking echocardiography. Global longitudinal strain; Left ventricular systolic function; child. We include publications in French and English between 19270 and 2020.

### Ethics and consent

After approval from the ethics committee of the main military hospital of Tunis (local committee for the protection of persons of the Military Hospital of Tunis (N°65/2022/CLPP/Military Hospital of Tunis ***)*** we included children under 18 years of age, regularly followed in the pediatric department, with homozygous SCA. For logistical reasons, the number of patients was limited to 30. Written informed consent was then obtained from the parents in view of the non-invasive nature of the parameters studied. Anonymity was respected during data entry.

## Results

### SCA group

*Demographics*

The mean age of our population was 12 ±4 years with extremes ranging from 3 to 17 years. The mean age of discovery of SCA was 2 years. Our population included 13 girls, 43.3% of the population, and 17 boys (56.7%). The average weight in our population was 35±14 kg, with an average height of 131±31 cm, or an average body surface area of 1.16±0.32 m ^2^.

*Clinical data*

Clinical examination data

Blood pressure measurement found a mean systolic blood pressure of 103±9 mmHg and a mean diastolic blood pressure of 68±9 mmHg. The mean heart rate was 93±8 cpm.

Clinical symptomatology and complications

The symptoms found in the SCA group and the complications reported are summarized in Table 1.

**Table 1.** Clinical symptomatology and complications in the SCA group (N=30).

	Number of patients (n)	Frequency (%)
**Dyspnea on exertion**	14	46.6%
**Chest pain**	9	30%
**Lipothymia**	3	10%
**Splenomegaly**	12	40%
**Vaso-occlusive crisis**	28	93.3%
**Osteonecrosis**	3	10%
**Splenic sequestration**	10	33.3%
**STROKE**	5	16.6%
**Splenectomy**	12	40%
**ATS** *	21	70%

* ATS=Acute Thoracic Syndrome.

*Biological data*

The average hemoglobin level in our patients was 8.6±0.5 g/dl. The mean ferritin level was 824±32 μg/l for a normal value between 7 and 140 μg/l.

Pharmacological treatment

The various medications received by our patients were penicillin V in 43%, hydroxycarbamin in 46%, deferoxamine in 16% and vitamin E in 96%. All patients were on folic acid.

### Comparative study

*Clinical and anthropometric data*

The comparison of the age of the children in the two groups did not show a statistically significant difference. The mean age in the SCA group was 12 years compared to 11 years in the C group (p=0.235). The sex ratio was 0.76 in the SCA group with a female predominance. In the C group, there was a male predominance with a sex ratio of 1.5 but this difference was not statistically significant (p=0.150).

Anthropometric data were comparable in both groups as shown in Table 2.

**Table 2.** Comparison of anthropometric data of the two groups.

	SCA group (N=30)	C group (N=30)	p
**Mean weight (kg)**	35±14.7	31.2±12.3	0.43
**Mean height (cm)**	131±31.8	138.5±24.6	0.521
**Body surface area Mean (m** ^**2**^ **)**	1.16±0.31	1.11±0.28	0.528

*Standard echocardiography data*

Morphological study of the left ventricle

The dimensions of the left ventricle in sickle cell children are summarized in Table 3.

**Table 3.** Comparison of the values of the morphological parameters of the LV in the two groups.

	SCA group (N=30)	C group (N=30)	p
**LVtdD** ^*^ **indexed (mm/m** ^**2**^ **)**	39.3±14.6	28.9±5.03	0.001
**ISW (mm)** ^**†**^	7.09±1.7	6.2±1.2	0.037
**LVM ind (g/m** ^**2**^ **)** ^**‡**^	98.7±34.2	62±16.5	<0.0001
**RWT** ^**§**^	0.29±0.03	0.39±0.05	0.035

* LVtdD=telediastolic diameter of the left ventricle.

^†^ ISW=interseptal wall.

^‡^ LVMind=indexed left ventricular mass.

^§^ RWT=Wall Relative Thickness.

Study of the LV systolic function

We measured the left ventricular ejection fraction (LVEF) by the Teicholtz method in both groups of patients. In SCA group, the mean LVEF was 58% with a minimum LVEF of 52% and a maximum of 77%. LVEF was maintained in all sickle cell patients. In time movement (TM) mode, we found a mitral annular plane systolic excursion (MAPSE of 14.6±3 mm in SCA group versus 13±3.3 mm in C group. In the LV tissue Doppler study, an LV S’ wave was measured at 9±2.6 cm/s in SCA group versus 8.7±1.7 cm/s in C group (p=0.685). The calculation of indexed cardiac output was comparable in both groups ( Table 4)

**Table 4.** Comparison of LV systolic function parameters between the two patient groups.

	SCA group (n=30)	C group (n=30)	p
**LVEF** ^*^ **(%)**	58±5.6	63.2±4.9	<0.01
**S’LV (cm/s)**	9±2.6	8.7±1.7	0.685
**MAPSE (mm)** ^**‡**^	14.6±3	13±3.3	0.429
**Cardiac output (l/min/m ^2^)**	3.93 ±2.1	4.08 ±3	0.464

* LVEF=left ventricular ejection fraction.

^‡^ MAPSE=Mitral annular plane systolic excursion.

.

*Study of longitudinal myocardial deformation using the speckle tracking technique*

The measurement of the left ventricular global longitudinal strain LVGLS showed a mean value of -21.2±3% in the SCA group.

The GLS was impaired in 46% of cases for a reference threshold of -20%.

**Figure 3.**
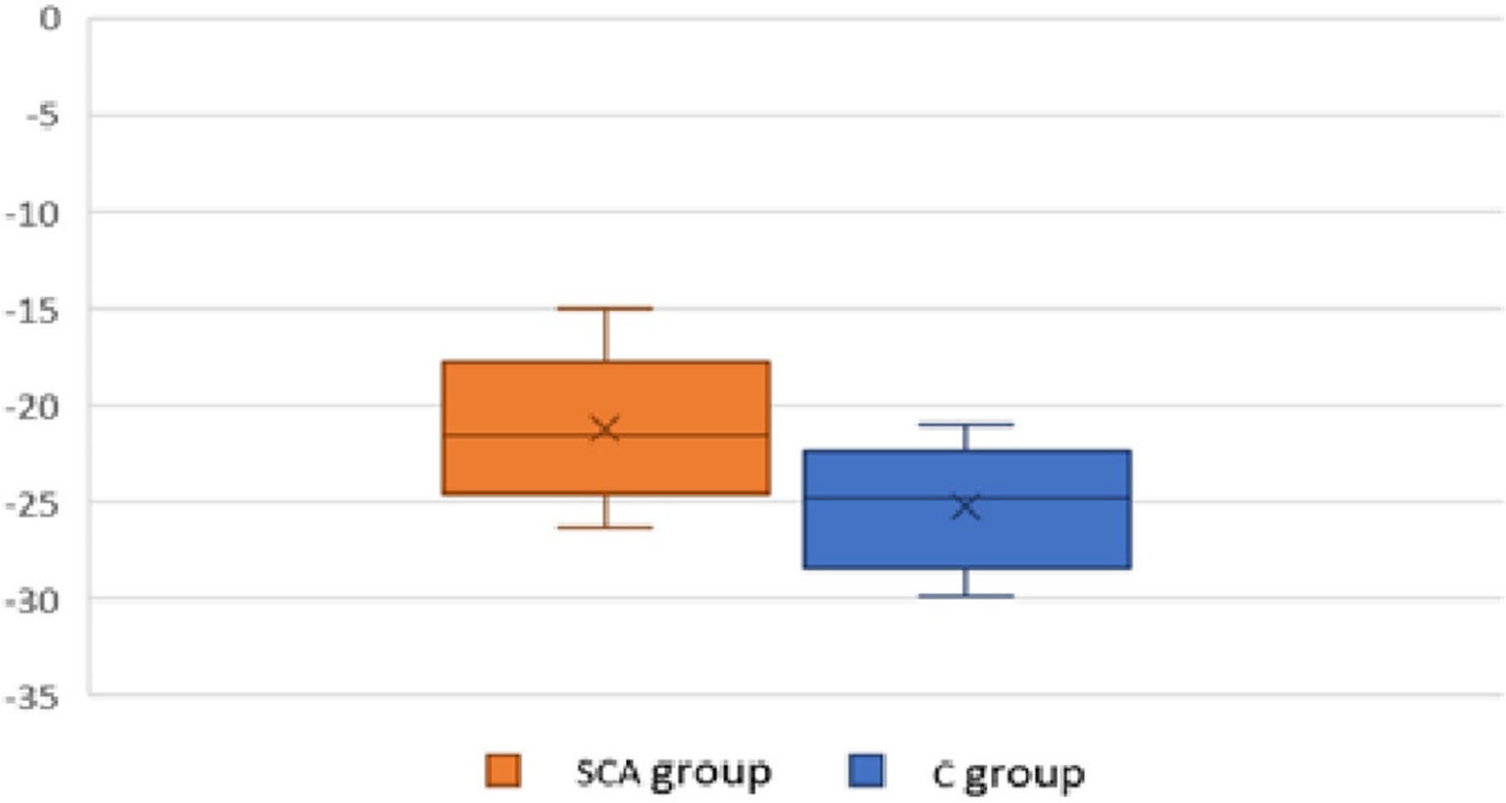
Comparison of the overall longitudinal LV strain in the two groups.

The comparative study between the two groups of patients showed a statistically significant difference with a mean value in SCA group of -21.22±3%, compared to -25.03±2.9% in C group (p<0.01) ( Figure 3).

Predictive factors for alteration of the global longitudinal strain of the LV

We found a p-value in the limit of significance between the occurrence of certain complications and the alteration of GLS such as osteonecrosis (p=0.051), splenomegaly (p=0.051, R=0.61). The occurrence of splenic sequestration (p=0.07, R=0.34) and dyspnea (p=0.075, R=0.38) were close to statistical significance. The influence of hydroxycarbamine treatment on GLS alteration was close to significance (p=0.075, R=-0.07). We found a statistically significant influence between GLS alteration and hydroxycarbamine dosage (p=0.043) ( Table 5).

**Table 5.** Correlation between clinical data and alteration of the global longitudinal strain of the LV in sickle cell patients.

Clinical data	Impaired GLS	Normal GLS	p
**Age at diagnosis (years)**	2.32±0.77	1.96±1	0.297
**VOC** ^**‡**^ **/year**	3.36±1.33	2.69±1.53	0.217
**ATS** ^*^	71.4%	68.8%	0.873
**STROKE**	21.4%	12.5%	0.513
**Osteonecrosis**	21.4%	100%	**0.051**
**Splenic sequestration**	50%	18.8%	**0.07**
**Dyspnea**	64.3%	31.3%	0.14
**Chest pain**	14.3%	18.8%	0.743
**Lipothymia**	14.3%	6.3%	0.464
**Number of Transfusion**	11±6	9±9	0.561
**Vitamin E treatment**	92.9%	100%	0.277
**Hydroxycarbamine treatment**	64.3%	31.3%	**0.07**
**Hydroxycarbamine dosage**	1000	800±273	**0.043**
**Splenectomy**	42.9%	37.5%	0.765
**Chelation**	21.4%	12.5%	0.513
**Splenomegaly**	50%	35.7%	**0.049**

^‡^ VOC=vaso-occlusive crisis.

* ATS=acute chest syndrome.

Biological data

We did not find any association between the alteration of the GLS and the biological data: hemoglobin level (p=0.261, r=-0.11), Ferritin level (p=0.734, r=-0.08).

Echocardiographic parameters

LV dilatation was correlated with impaired LV GLS (p=0.04; R=0.27). Furthermore, LV hypertrophy was also associated with Strain impairment with a statistically significant p for both indexed LV mass (p=0.007; r=0.41) and posterior wall thickness (p=0.016; R=0.33).

## Discussion

We performed a comparative study in which we were interested in the comparison of several morphological and functional echographic parameters of the left ventricle between a first group (SCA group) of 30 children under 18 years of age followed up for major sickle cell syndrome, and a second group (C group) of 30 healthy controls. The two groups of patients were comparable for sex, age, and body surface area. At the end of the study, we found some morphological differences in the left ventricle between the two groups.

Our results mainly support the use of 2D strain in the assessment of left ventricular function in our population. The study of the myocardial strain by speckle tracking technique allowed us to objectivate a statistically significant difference in the GLS between the two groups of patients (p<0.01) despite a preserved value of the LVEF.

The analytical study of the data collected allowed us to show a significant correlation between this alteration in strain and certain clinical and echographic parameters.

### Dilatation of the left ventricle

In our study, we noted a significantly greater dilation of the LV in the SCA group compared to the C group. Indeed, the chronic anemia due to hemolysis in this disease induces an increase in cardiac output with a minimal increase in heart rate. The systolic ejection volume (SEV) then increases, resulting in significant dilation of the LV. ^5^

In addition, this chronic intravascular hemolysis is often associated with pulmonary vaso-occlusive events, which contributes to oxygen desaturation of the blood and increased cardiac output to accommodate this hypoxemia.

The degree of LV dilatation has been described as proportional to the degree of anemia in some studies. ^6^^,^^7^ However, increased cardiac output in non-sickle cell anemia usually occurs when the hemoglobin level is less than or equal to 7 g/100 ml. A few studies have been carried out using hemodynamic measurements of cardiac output in homozygous sickle cell disease and have confirmed the existence of a significant increase in resting cardiac output in most patients, even for hemoglobin levels of 9 to 10 g/100 ml. Thus, for the same hemoglobin level, resting cardiac output was higher in sickle cell patients than in subjects with chronic anemia of other etiologies. ^1^ This is probably due to the oxygen desaturation of arterial blood, due to the decreased affinity of hemoglobin S for oxygen, but even more so to the formation of left-sided intra pulmonary shunts due to vaso-occlusive phenomena. ^8^

This increase in cardiac output has long been incriminated as the primum movens of cardiac damage in sickle cell patients. It has been demonstrated in several studies by measurement of cardiac output by transthoracic ultrasound and by invasive hemodynamic measurements and has often been associated with an increase in morbidity and mortality in these patients. ^9^

### Left ventricular hypertrophy

In the course of this work, we noted significantly more left ventricular hypertrophy in the SCA group compared to the C group. This LV remodeling was rather eccentric in our population. Indeed, to compensate for the increase in end-diastolic diameter, and therefore the increase in parietal stress, there is an increase in left ventricular mass. According to Laplace’s law, ventricular wall stress is directly proportional to LV pressure and diameter, and inversely proportional to wall thickness. In addition, iron overload may develop during transfusions and have an impact on the evolution of this myocardial hypertrophy. ^10^

### Left ventricular systolic function

LV systolic function was assessed by several parameters in this study. LVEF measurement by the Teicholtz method was maintained in all patients but the mean was significantly lower in the SCA group. No significant differences were found for the rest of the parameters, as well as for cardiac output.

This result has been confirmed by several studies and meta-analyses. A meta-analysis published in 2013 included 19 studies involving 841 subjects with sickle cell disease and 554 controls. In this meta-analysis no statistically significant difference was found in LVEF and RF between patients and controls with p values of 0.76 and 0.28 respectively. ^11^

Lamers *et al.* studied 57 children with sickle cell disease compared to 50 healthy children. They studied myocardial contractility using parameters that depend on loading conditions, such as shortening fraction (SF), which was lower in sickle cell children, but within the normal range for age. ^12^

Wali *et al.* studied LV systolic function in 40 children with sickle cell disease compared to 25 controls. No statistically significant differences were reported in the study of LV systolic function. ^13^

The various studies that have assessed LV systolic function in sickle cell patients have concluded that myocardial contractility is impaired based on parameters independent of loading conditions. The load-dependent measures would be compensated for in early life and would progressively deteriorate with age. ^14^ Consistent with these data, assessment of LV systolic function by measuring MAPSE in TM mode and LV S’ wave velocity by tissue Doppler at the mitral annulus found no significant difference in our pediatric population compared with the control group.

The mechanism of cardiomyopathy in sickle cell disease is twofold: chronic left ventricular volume overload due to anemia associated with repeated ischemic events due to thrombosis of the coronary microcirculation. This leads to dilation and myocardial remodeling which will evolve towards impairment of LV systolic function. However, the interpretation of the usual systolic function parameters must be done with caution under abnormal loading conditions in sickle cell patients, and the demonstration of this impairment requires ultrasound parameters that are not dependent on loading conditions.

*Value of speckle tracking in the study of systolic function in sickle cell patients*

In this work, the LVGLS was significantly more impaired in sickle cell patients than in controls. This result has been found in some studies. ^13^^,^^15^

Indeed, global longitudinal strain correlates better with myocardial fibrosis than LVEF and its interest in these patients lies mainly in its sensitivity to detect early alterations in systolic function despite a preserved LVEF. ^16^^,^^17^

Indeed, the LV dysfunction reflected by the altered strain in sickle cell patients could be the result of myocardial ischemia, fibrosis or iron deposition in the myocardium or ventricular hypertrophy which could be associated at the beginning with a preserved LVEF value. ^18^ Furthermore, it is recognized that in ischemic or non-ischemic dilated heart disease, alterations in GLS generally correlate well with LVEF and precede it during the course of the disease. This correlation would be particularly useful in practice for monitoring cardiomyopathy in sickle cell patients as it could provide a reproducible estimate of LVEF measured in Simpson biplane and could thus be proposed as a reproducible tool to reduce inter- and intra-observer variability in the study of myocardial strain and thus improve patient management by identifying myocardial damage at a sub-clinical stage. ^19^

### Predictive factors of Strain alteration in sickle cell disease

After analysis of the clinical data in our sickle cell patients, the predictive factor of an alteration of the LVGLS found in this work was mainly the treatment with hydroxycarbamine. A trend towards significance was noted for the occurrence of complications including osteonecrosis, splenomegaly, and splenic sequestration.

Indeed, hydroxycarbamine is a particularly important molecule in the treatment of sickle cell disease by stimulating the synthesis of foetal hemoglobin which plays a protective role in these patients. A study published by Sachdev *et al*. in 2017 ^20^ demonstrated a significant improvement in coronary analysis of the ultrasound parameters and revealed a correlation between alteration of LV GLS and LV dilatation and hypertrophy. LV dilatation was correlated with GLS alteration in this work. These echographic findings found in several series ^21^^,^^22^ derive from the pathophysiological mechanisms of cardiac involvement in sickle cell disease and could be explained mainly by the progression of myocardial fibrosis and diastolic dysfunction which constitute an independent prognostic factor in these patients. ^20^ The GLS study could thus be proposed as an endpoint for the prospective study and ultrasound follow-up of sickle cell patients, but its clinical consequences remain to be evaluated by large-scale studies. ^23^

Recent studies of the genetic basis of transmission of sickle cell disease have identified certain genotypes associated with cardiovascular disease. ^24^ It is therefore necessary to couple the study of echocardiography and strain with genetic analyses to better study the different phenotypes and forms of this disease, to eventually guide screening, follow-up, and genetic counselling in these patients.

Finally, the exact incidence of cardiomyopathy in sickle cell disease is still unclear. ^25^^,^^26^

The interpretation of our results was further limited by the lack of recognized standard norms of strain in the pediatric population and by the complexity of using the reference values of the Z score, in the morphological study of the LV, which remains little used in current practice, but which may have reservations about our results.

In this work, the LVGLS was significantly more impaired in sickle cell patients than in controls. However, larger scale studies are therefore required to better study the role of LV strain in the evaluation of myocardial damage in children with sickle cell disease and in the estimation of the risk of progression to heart failure in these patients, in order to guide therapeutic management. It seems necessary to estimate the cardiovascular risk of this population and to propose management algorithms that may include the study of myocardial deformation to reduce the risk of sudden death and the progression to heart failure.

## Consent

Written informed consent was obtained from the parents.

## Author contributions

Each author has contributed to this work as follows:

**Sarra Chenik:** Conceptualization, Data Curation, Methodology, Resources, Validation, Writing – Original Draft Preparation, Writing – Review & Editing

**Aymen Noamen:** Data Curation, Methodology, Resources, Validation, Writing – Original Draft Preparation

**Abyr Bouslimi:** Data Curation, Methodology, Supervision, Validation, Writing – Original Draft Preparation, Writing – Review & Editing

**Houaida Mahfoudhi:** Supervision, Validation, Visualization, Writing – Review & Editing

**Sadok Hannachi:** Conceptualization, Data Curation, Methodology, Resources, Supervision, Validation, Writing – Original Draft Preparation, Writing – Review & Editing

**Hager Barakizou:** Resources, Validation, Visualization

**Islem Mejri:** Validation, Visualization, Writing – Review & Editing

**Tasnim znegui:** Visualization

**Wafa Fehri**: Supervision, Validation, Visualization, Writing – Review & Editing

## Data Availability

figshare: Echocardiographic evaluation in children with sickle cell anemia: contribution of 2D strain, https://doi.org/10.6084/m9.figshare.20949031.v4. ^27^ Data are available under the terms of the Creative Commons Attribution 4.0 International license (CC-BY 4.0).
