# Evaluation of left ventricular systolic function in children with sickle cell anemia: contribution of 2D strain

**DOI:** 10.12688/f1000research.125345.2

**Published:** 2022-12-05

**Authors:** Sarra Chenik, Aymen Noamen, Abyr Bouslimi, Houaida Mahfoudhi, Sadok Hannachi, Hager Barakizou, Islam Mejri, Tasnim Znegui, Wafa Fehri

**Affiliations:** 1 Cardiology department, Military Hospital of Tunis, Tunis, Tunisia; 2 Cardiology department, Nantes Hospital,France,, Nantes, France; 3 Pediatric department, Military Hospital of Tunis, Tunis, Tunisia; 4 Pneumology department, Military Hospital of Tunis, Tunis, Tunisia

**Keywords:** sickle cell anemia; heart disease; echocardiography; Speckle tracking echocardiography. Global longitudinal strain; Left ventricular systolic function; child.

## Abstract

Background:

Children with sickle cell anemia (SCA) are at an increased risk of cardiovascular complications. The aim of this study was to assess the role of speckle tracking echocardiography in detecting subclinical myocardial damage in children with SCA.

Methods:

A cross-sectional case–control study was conducted at the echocardiography laboratory of the military hospital of Tunis between July and December 2018. Thirty patients with SCA were included. A control(C) group including 30 normally developing children was selected and matched to the SCA group by sex and age. We compared between the two groups: conventional echocardiographic parameters including cardiac output, left ventricular ejection fraction (LVEF), thickness and the global longitudinal strain (GLS). The echocardiographic measurements were indexed according to body surface area. The left ventricular (LV) GLS association with clinical characteristics and echocardiographic parameters were also evaluated.

Results:

Patients and controls were matched for age and sex: the mean age was (11± 2years) in SCA group versus (12± 1 years) in C group with a sex ratio of (1.31 versus 1.27, respectively). Body surface area was comparable. LV hypertrophy and dilation were revealed in the SCA group, whereas measurements were normal in the C group. No significant differences were observed for cardiac output (p=0.4). LVEF were preserved in both groups. However, two-dimensional (2D) LVGLS was impaired in 46% of SCA group (n=14) with mean value of (-21%±3.07 vs -25%±2.98; p<0.01).In SCA group, impaired LVGLS was significantly associated with LV mass (r = – 0.399, p<0.01), LV tele diastolic diameter(r= -0.419, p<0.01) and left atrial volume (r= - 0.399, p< 0.04). In multivariate analysis, LV mass was the only independent factor.

Conclusions:

In the present study, LVGLS measurement revealed subclinical LV systolic impairment in patients with SCA. Therefore, 2D strain could be beneficial to detect the natural history of LV dysfunction in SCA.

## List of abreviations

2D: Two-dimensional

C: Control

DTI: Pulsed Doppler tissue

GLS: Global longitudinal strain

IVST: Interventricular septal wall thickness

LV: Left ventricular

LVEDD: Left ventricular end-diastolic diameter

LVEF: Left ventricular ejection fraction

LVESD: Left ventricular end-systolic diameter

LVGLS: Left ventricular global longitudinal strain

LVM: Left ventricular mass

MAPSE: Mitral annular plane systolic excursion

PWT: Posterior wall thickness

S’: Systolic mitral annulus velocity

SCA: Sickle cell anemia

TM: Time Movement (TM)

TTE: Transthoracic echocardiography

## Introduction

Sickle cell disease or sickle cell anemia (SCA) is an autosomal recessive genetic disease linked to a hemoglobin abnormality leading to the deformation of red blood cells. ^1^ The disease affects more than 50 million people worldwide, particularly in sub-Saharan Africa and the Mediterranean region. ^2^ Globally, hemoglobin disorders are responsible for about 3.4% of death among children under 5 years old. ^2, 3^

The prognosis of this serious chronic disease depends on the occurrence of complications, particularly cardiovascular diseases. However, these complications usually occurred in adulthood, largely due to a lack of regular cardiological follow-up during childhood. ^4^ The development of cardiac complications in SCA are multifactorial including anemia related chronic volumetric overload, endothelial dysfunction, altered microcirculation, and myocardial iron overload. All these mechanisms contribute to severe clinical manifestations, ranging from pulmonary arterial hypertension to left ventricular diastolic dysfunction and dilated or hypertrophic cardiomyopathy. ^5^ Nowadays, with the advent of new echocardiographic techniques such as two-dimensional (2D) strain, it is possible to detect systolic and diastolic dysfunction earlier than with conventional echocardiography. ^6^

The advent of 2D strain has provided early detection of cardiac damage in many chronic diseases and has increasingly become essential to stratify prognosis. In fact, it is a new technique that studies myocardial motion by tracking speckles, which are acoustic markers of the myocardium. ^6^ Local tissue motion is represented by the geometric displacement of each speckle. Several software packages have been developed to allow temporal and spatial processing of the image obtained by the 2D strain. ^6^

The aim of the present study was to assess the contribution of 2D strain to the detection of subclinical left ventricular (LV) myocardial damage in children with SCA.

## Methods

### Study design

A cross sectional case-control study was conducted in the cardiology department at the Military Hospital of Tunis between July and December 2018.

### Study population

• *Samplesize calculation:*
  Knowing that 2.4% of patients followed up on at the pediatric department were referred for pediatric cardiology consultation during the study period, it was deemed that a sample size of 35 patients, calculated using a predictive formula, would be required to achieve statistical significance (power: 0.8; alpha: 0.05). ^7^
• *SCA group:*
  Inclusion and non-inclusion criteria: The SCA group included children (2-18 years) followed up at the pediatric department of the military hospital of Tunis for homozygous SCA during the study period. Patients with a history of heart disease or those who had undergone cardiac surgery were not included.
  Exclusion criteria: The patients with poor echogenicity on transthoracic echocardiography (TTE) and those who were lost to follow-up during the study period were excluded.
• *Control group:*
  For the control (C) group, we selected 30 children with no history of cardiovascular or respiratory pathology or presenting anemia at the time of the study. Those children were hospitalized in the pediatric department and referred to a pediatric cardiologist for assessment of a heart murmur or for exploration of atypical chest pain. All the control patients had normal echocardiography. Then, the control group was matched with the SCA group based on age and gender.

### Data collection

Data were collected by a single physician (? For example SC or AN …) using the patient information sheet. Parents accepted to participate in the study and informed consents were given. They also accepted that their children underwent an echocardiographic examination since it is noninvasive.

**Figure 1.**
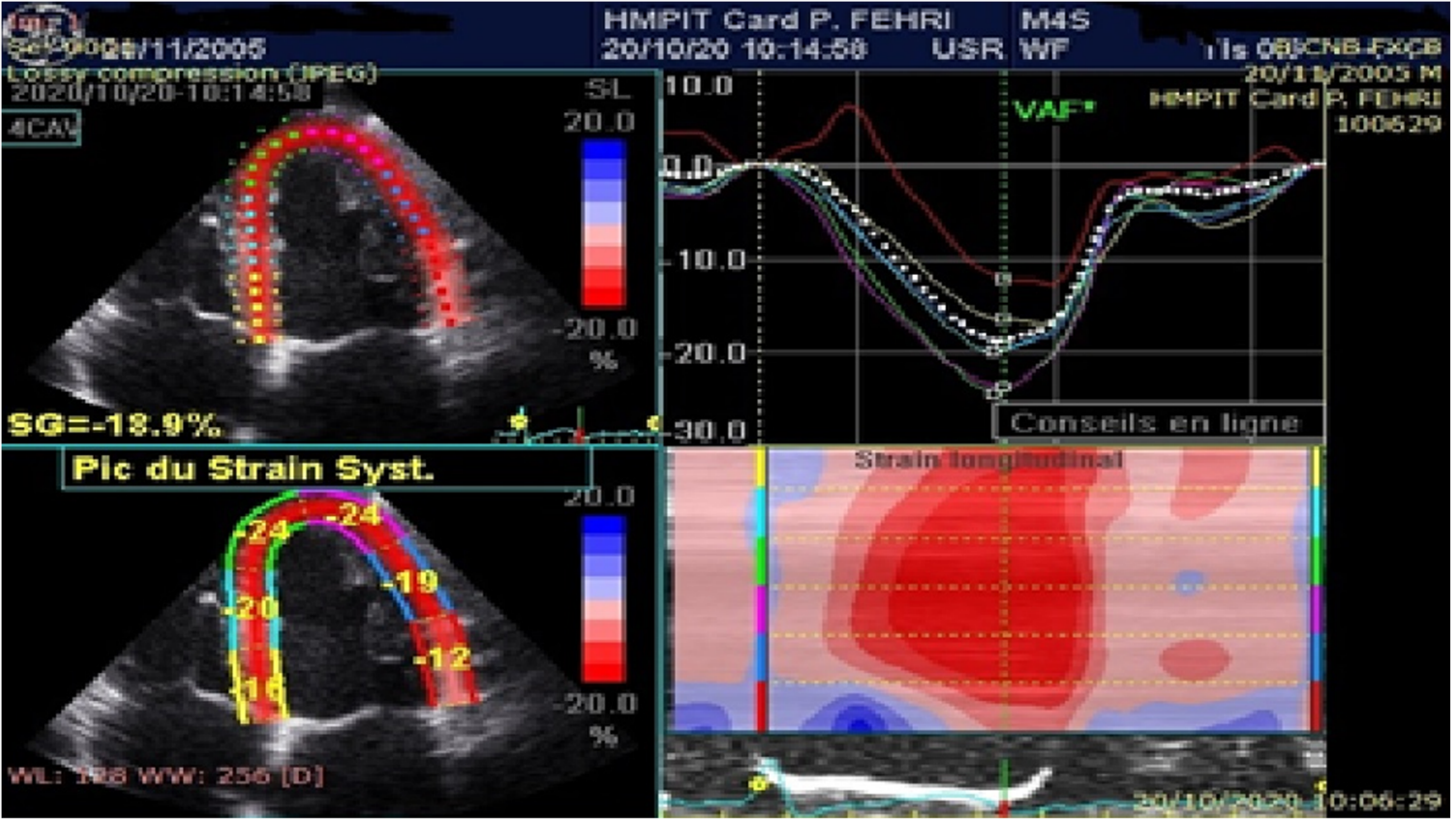
Analysis of longitudinal deformations of the apical incidence of 4 cavities in a healthy population.

**Figure 2.**
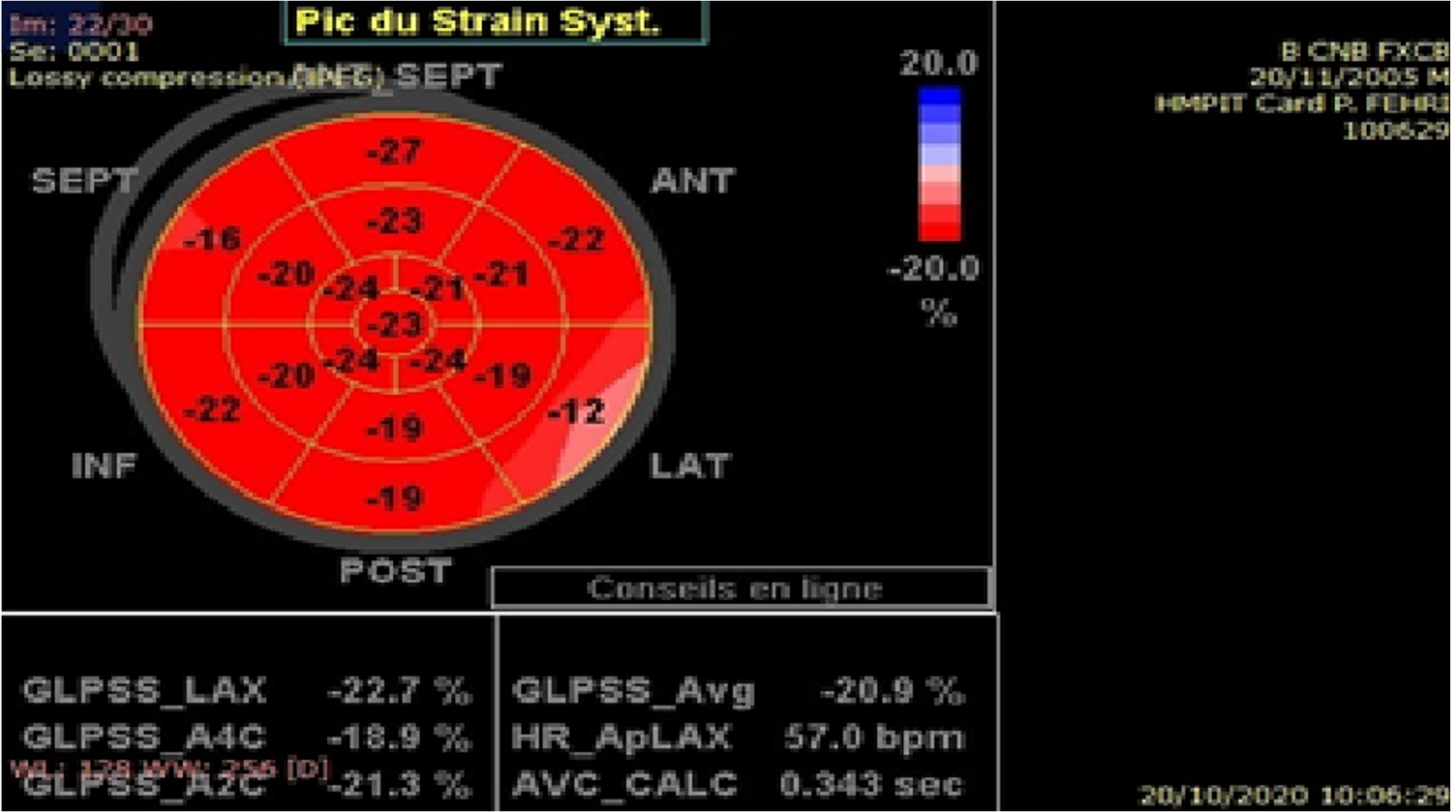
Segmental and global left ventricular longitudinal strain in a healthy population.

• *Clinical and anthropometric data*
  Data collected were age, sex, medical history, age of onset of disease, hemoglobin level, rate of transfusion, complications, and current treatment. The height was measured with a standing stadiometer (Seca 217), barefoot or in socks, in a standing position, heels joined, well balanced, and back straight. Weight (±1 kg) was measured with a digital scale (Tanita TBF-300 body composition analyzer), and then body surface area (m ^2^) was calculated. ^8^
• *Echocardiography*
  TTE was performed in all patients by a single experienced pediatric cardiologist (same who ? initials) using a vivid E7 ultrasound system (GE Healthcare, Horten, Norway) with a S5-1 probe according to the guidelines of the American Society of Echocardiography and the European Association of Cardiovascular Imaging. ^9^
  All echocardiographic examinations were analyzed by the same cardiologist who was not aware about the collected clinical data (blind analysis). Measurements were performed in the parasternal long axis, parasternal short axis, and apical views (two, three, and four chamber views). An electrocardiogram with a velocity scale between 12 and 20 cm/sec was performed to avoid aliasing.
  Each incidence was performed so that the angle between the examined myocardial wall and the ultrasound beam did not exceed 30 degrees. All echocardiographic data were stored on a central memory unit, allowing post processing and adjustment of measurements, including pulsed Doppler tissue imaging (DTI), measured, and averaged over 3 to 5 continuous cardiac cycles.
  Left ventricular end-diastolic diameter (LVEDD), left ventricular end-systolic diameter (LVESD), interventricular septal wall thickness, and posterior wall thickness (PWT) were measured with 2D targeted M-mode tracing. The left ventricular mass (LVM) was estimated using the Devereux formula. ^10^
  Left ventricular ejection fraction (LVEF) was calculated using Simpson's biplane method of discs, and systolic mitral annulus velocity (S’) was assessed using DTI in 2D mode of the mitral annulus in the four-chamber view. ^10^
• *Strain analysis by speckle-tracking echocardiography:* myocardial deformation assessment
  Speckle tracking analysis was performed offline on views of the four apical chambers, the apical long axis, and the apical two chambers that were previously stored in Digital Imaging and Communications in Medicine format. Analysis of echocardiographic images was performed offline using Echo PAC (GE Medical Systems, Norway). Three cardiac cycles were recorded in cine loop format at a frame rate between 50 and 80 frames per second as 2D grayscale views.
  One end systolic frame is selected by the operator to perform the manual tracing of the endocardial border of the LV. This tracing is then used by the software to create a region of interest. The myocardium is automatically divided into segments according to the standard 16-segment model of the LV. ^11^ The quality of myocardial tracking was visually checked in real time and then manually corrected to ensure optimal tracking. The software then tracks the deformation of the myocardium during the cardiac cycle to calculate peak systolic segmental strain. The global longitudinal strain (GLS) is determined as the average of the segmental strains ( Figures 1, 2). The mean LVGLS estimated in healthy children at -20.2% (95% CI: -19.5%, -20.8%) was used as the cutoff level. ^12^

### Statistical analysis

All statistical analyses were conducted using SPSS 25.0 software (IBM SPSS Inc., Chicago, Illinois, USA). Quantitative variables were tested for normal distribution using the Shapiro-Wilk tests and then expressed as medians and interquartile. Qualitative variables were expressed as numbers and percentages. For the case-control study, the Mann-Whitney test was used to compare categorical data. The optimal cutoff value of GLS in the SCA group was determined based on receiver-operator characteristics (ROC) curve analysis.

A linear regression model was used to evaluate the relationship between impaired LVGLS and clinical and echocardiographic measurements. Pearson correlation analysis and Spearman rank correlation analysis were performed to assess dependence.

The multivariate linear regression for assessing independent correlations in the impaired GLS was performed by including significant variables from the univariate model. A p<0.05 indicated statistical significance.

### Literature review

For bibliographic research, we used PubMed through MESH research based on the following keywords: Sickle cell anemia, heart disease, echocardiography, speckle tracking echocardiography, global longitudinal strain, left ventricular systolic function, and child. We included publications in French and English between 1970 and 2020.

### Ethics and consent

The study was approved by the ethics committee of the military hospital in Tunis. Written informed consent was collected from the parents. Anonymity was respected during data treatment.

## Results

**Figure 3.**
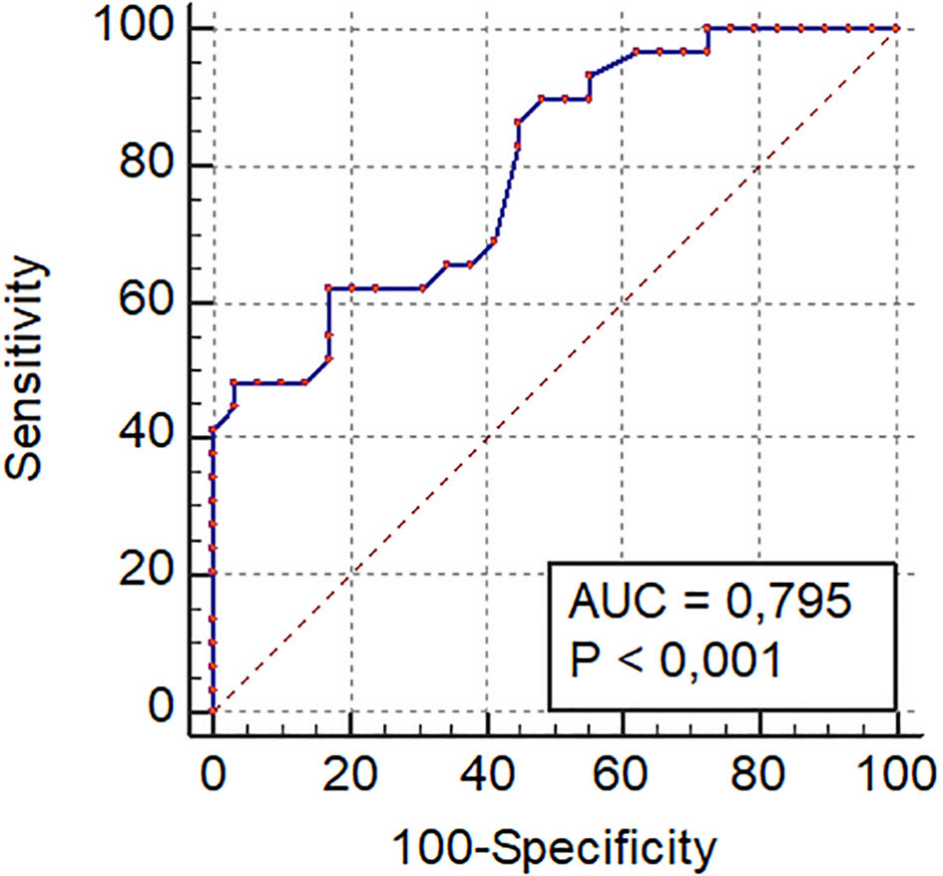
Curve receiver operating characteristic (ROC) of left ventricular strain.

**Figure 4.**
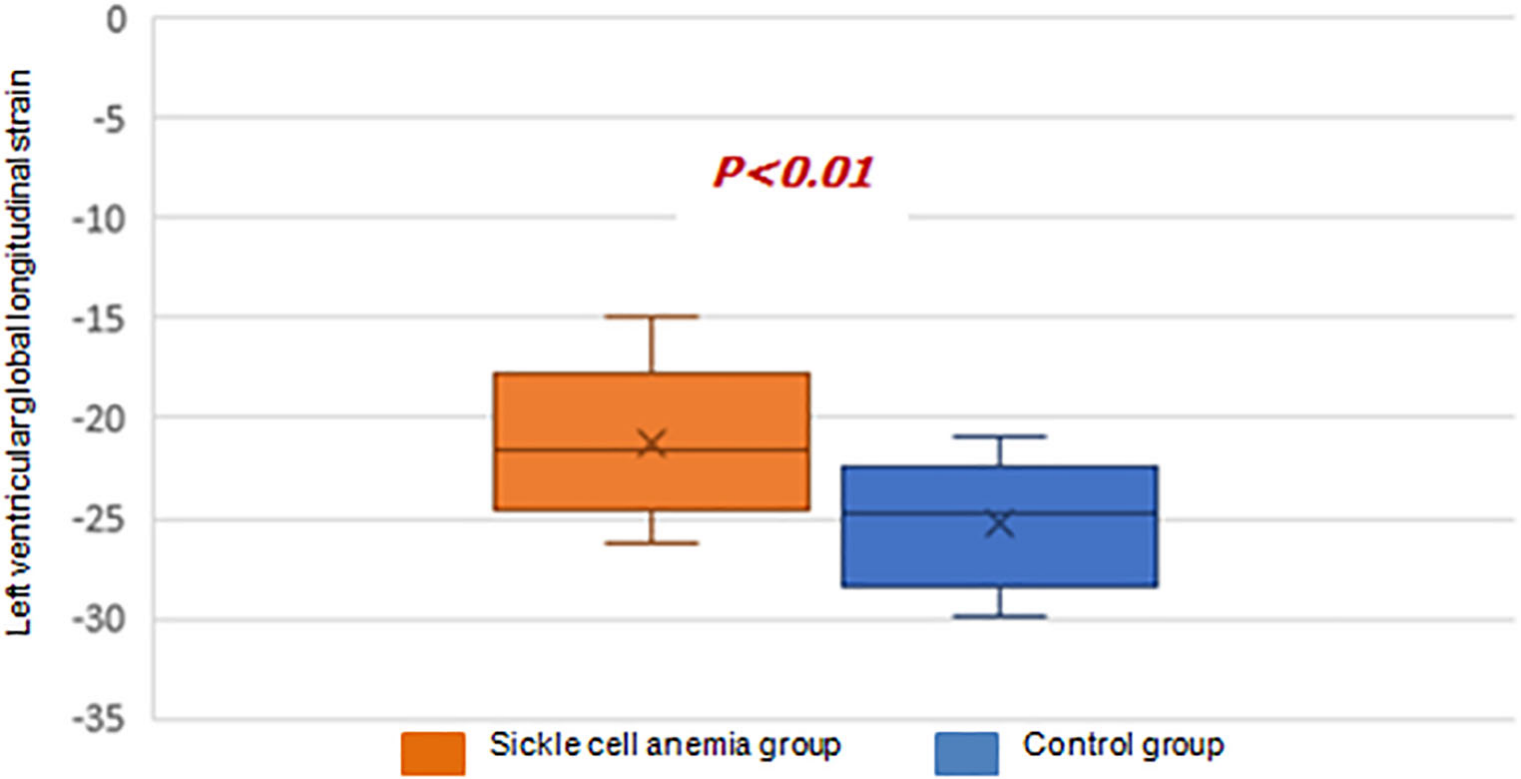
Comparison of the left ventricular global longitudinal strain in the two groups.

• *Baseline characteristics of the two groups*
  Patients and controls were matched for age and sex ( Table 1). The mean age of children with SCA was 12±4 years including 13 girls (43%) and 17 boys (57%). The mean age of the control group was 11±3 years (18 boys and 12 girls). In terms of body surface area, there was no statistically significant difference between the two groups.
  The average hemoglobin level in the SCA group was 8.6±0.5 g/dl. The mean serum ferritin value was 824±32 μg/l. The treatment received by SCA children included intravenous penicillin in 43% (n=13), Hydroxycarbamide in 46% (n=14), Deferoxamine in 16% (n=5) and vitamin E in 96% (n=29) of the patients. All patients received folic acid.
  The symptoms of the SCA group and reported complications are summarized in Table 2.
• *Echocardiographic measurements*
  Morphological characteristics and LV systolic function are summarized in Table 3. LV dimensions and mass were significantly greater in the SCA group than in the C group. However, the LVEF measured by the Teicholtz method, was preserved in both groups. A significant difference in the mean LVEF value was noted: 58±12% for the SCA group versus 63±5% for the C group.
  No significant differences were revealed between the 2 groups for mitral annular plane systolic excursion (MAPSE) and LV systolic mitral annulus velocity (S') wave measurements. The calculation of the indexed cardiac output was comparable in both groups.
• *Study of longitudinal myocardial deformation using the speckle tracking technique*
  According to this study, the optimal cutoff value of GLS was -21.3% (AUC 0.79, 95% CI [0.68–0.90], p<0.001). The sensitivity, specificity, positive predictive value, and negative predictive value were 48%, 82%, 96%, and 65%, respectively, for predicting altered LV contractile function ( Figure 3).
  In the SCA group, LVGLS was estimated at -21.2±3%. It was significantly reduced (-20%) in 46% of the children (n=14), whereas it was significantly higher (-25.03±2.9%) in the C group with no decreased value ( Figure 4).
• *Univariate and multivariate analyses of factors associated with abnormal LVGLS*
  In the SCA group, impaired LVGLS was significantly associated with left ventricular mass (LVM), LV tele diastolic diameter, and left atrial volume ( Table 4).
  No significant correlations were noted between the altered LVGLS, clinical symptoms, complications, and hemoglobin level. The multivariate analysis found a correlation between LVM and impaired GLS (b=-0.082, p<0.001).

**Table 1.** Comparison of baseline characteristics of the two groups.

Variable	SCA group	C group	p-value
**Age (years)**	12±4	11±3	0.23
**Sex ratio**	0.76	0.67	0.15
**Mean weight (kg)**	35±14.7	31.2±12.3	0.43
**Mean height (cm)**	131±31.8	138.5±24.6	0.521
**Mean body surface area (m** ^**2**^ **)**	1.16±0.31	1.11±0.28	0.528
**Hemoglobin (g/dl)**	8.5±0.5	12.8	**0.001** *

SCA: Sickle cell anemia group, C group: Control group.

* p<0.05.

**Table 2.** Clinical findings and complications in the SCA group (N=30).

	Number of patients (n)	Frequency (%)
**Dyspnea on exertion**	14	47%
**Chest pain**	9	30%
**Lipothymia**	3	10%
**Complications:**		
**Splenomegaly**	12	40%
**Vaso-occlusive crisis**	28	93%
**Osteonecrosis**	3	10%
**Splenic sequestration**	10	33%
**Stroke**	5	16%
**Splenectomy**	12	40%
**Acute thoracic syndrome**	21	70%

SCA: Sickle cell anemia.

**Table 3.** Comparison of LV morphological parameters and LV systolic function in the two groups.

	SCA group (N=30)	C group (N=30)	P
**LVtdD indexed (mm/m** ^**2**^ **)**	39.3±14.6	28.9±5.03	**0.001** *
**ISW (mm)**	7.09±1.7	6.2±1.2	**0.037** *
**LVM ind (g/m** ^**2**^ **)**	98.7±34.2	62±16.5	**<0.0001** *
**RWT**	0.29±0.03	0.39±0.05	**0.035** *
**LVEF (%)**	58±5.6	63.2±4.9	<0.01
**S’LV (cm/s)**	9±2.6	8.7±1.7	0.685
**MAPSE (mm)**	14.6±3	13±3.3	0.429
**Cardiac output (l/min/m ^2^)**	3.93±2.1	4.08±3	0.464

SCA: Sickle cell anemia group, C group: Control group, LVtdD: Telediastolic diameter of the left ventricle, ISW: interseptal wall, LVMind: Indexed left ventricular mass, RWT: Wall Relative Thickness, LVEF: Left ventricular ejection fraction, S’: Systolic mitral annulus velocity in DTI, MAPSE: Mitral annular plane systolic excursion.

* p<0.05.

**Table 4.** Echocardiographic parameters according to impaired LVGLS in sickle cell anemia patients.

Echocardiographic parameters	Impaired GLS	
	R	P
**LVtdD**	-0.419	**0.001** *
**LVM**	-0.414	**0.001** *
**Left atrial volume**	-0.399	**0.004** *
**LVEF**	0.226	0.089
**PAPS**	-0.389	**0.003** *
**Em**	0.156	0.427
**Am**	0.575	**0.000** *
**E/Am**	0.060	0.768

GLS: Global longitudinal strain, LVM: Left ventricular mass, LVtdD: Telediastolic diameter of the left ventricle, LVEF: Left ventricular ejection fraction, PAPS: Systolic pulmonary artery pressure, Am: Mitral A wave, Em: Mitral E wave.

* p<0.05.

## Discussion

The main finding of the present study was that performing 2D speckle echocardiography in SCA patients may allow early detection of subclinical left ventricular morphological modifications such as hypertrophy and dilation. Even though both groups had preserved LVEF, the systolic function as measured by 2D speckle tracking imaging was impaired in 46% of the SCA group.

In fact, the greater dilation of the LV in the SCA group could be explained by several mechanisms. First, the chronic anemia due to hemolysis leads to an increase in cardiac output, systolic ejection volume, and baseline heart rate. All these contribute to a significant dilation of the LV. ^13^ In addition, the chronic intravascular hemolysis is commonly associated with vaso-occlusive event that also results in hypoxia and further increase in cardiac output. ^13^ In some studies, the degree of LV dilation has been described as proportional to the degree of anemia. ^14^ However, increased cardiac output in non-sickle cell anemia usually occurs when the hemoglobin level is less than or equal to 7 g/100 ml. A few studies have been performed using hemodynamic measurements of cardiac output in homozygous SCA and have confirmed the existence of a significant increase in resting cardiac output in most patients, even for hemoglobin levels of 9 to 10 g/100 ml. Thus, for the same hemoglobin level, resting cardiac output was higher in sickle cell patients than that of persons with chronic anemia from other etiologies. ^2^ This is most likely due to hypoxemia caused by hemoglobin S's decreased affinity for oxygen and intrapulmonary shunts caused by vaso-occlusive crisis. ^15^ Therefore, this increase in cardiac output has long been implicated in the prime cause of cardiac damage in sickle cell patients. It has been also associated with morbidity and mortality in these patients. ^16^

This study has also revealed features of left ventricular eccentric hypertrophy in SCA patients. The LVM may expand in the setting of SCA to accommodate the increase in tele diastolic diameter of LV. In addition, iron overload may develop during transfusions and boost myocardial development. ^17, 18^

In this study, LV systolic function was preserved in all patients with a decreased mean value of LVEF in the SCA group. No significant differences were found for the rest of parameters, including cardiac output value. This result was in line with several studies and meta-analyses. ^19, 20^ For example, no significant difference in LVEF was also observed in a meta-analysis involving 841 patients with SCA and 554 controls. ^19^ Otherwise, Lamers et al. have reported a decreased in fractional shortening in SCA children while studying myocardial contractility. ^21^ Several studies that have assessed left ventricular systolic function in sickle cell patients have concluded that myocardial contractility was impaired independently of left ventricular preload and after load. The load-dependent parameters would be compensated at early stage and would progressively deteriorate with age. ^22^ Chronic left ventricular volume overload and repeated ischemic events represent the main mechanisms of cardiomyopathy in SCA patients. It leads to myocardial dilation and remodeling that progressively impair left ventricular systolic function overtime. ^23^

The present study revealed that the LVGLS was significantly impaired in sickle cell patients compared to controls. Indeed, LVGLS is a more specific predictor of myocardial remodeling than LVEF. It is extremely sensitive in detecting early systolic function impairment despite a preserved LVEF. ^24, 25^ In fact, altered strain in sickle cell patients could be the result of myocardial ischemia, fibrosis, myocardial iron deposition, and ventricular hypertrophy which could be associated with a preserved LVEF during the early stage of the disease. Therefore, LVGLS could indicate the progression to cardiac disorders and myocardial damage at a subclinical stage. ^26^

In line with this study result, Sachdev et al. described a correlation between impaired LVGLS and LV dilation and hypertrophy. ^27^ Thus, the GLS seems to bea useful tool for the follow-up of sickle cell patients. ^28^ Recent genetic studies in SCA have identified certain genotypes associated with cardiovascular disease. ^29^ Therefore, it is maybe important to couple echocardiography with genetic analyses in order improve the follow-up and long-term prognosis of SCA patients. ^30^

## Conclusion

This study primarily supports the use of 2D strain in the assessment of LV function in SCA. However, the interpretation of these results was limited by the absence of recognized standard norms of strain in the pediatric population and by the complexity of using Z-score reference values in the morphological analysis of the LV. Larger scale studies are therefore required to improve the role of LV strain for early evaluation of myocardial damage in children with SCA and to predict the risk of progression to heart failure in these patients.

## Consent

Written informed consent was obtained from the parents.

## Author contributions

Each author has contributed to this work as follows:

**Sarra Chenik:** Conceptualization, Data Curation, Methodology, Resources, Validation, Writing – Original Draft Preparation, Writing – Review & Editing

**Aymen Noamen:** Data Curation, Methodology, Resources, Validation, Writing – Original Draft Preparation

**Abyr Bouslimi:** Data Curation, Methodology, Supervision, Validation, Writing – Original Draft Preparation, Writing – Review & Editing

**Houaida Mahfoudhi:** Supervision, Validation, Visualization, Writing – Review & Editing

**Sadok Hannachi:** Conceptualization, Data Curation, Methodology, Resources, Supervision, Validation, Writing – Original Draft Preparation, Writing – Review & Editing

**Hager Barakizou:** Resources, Validation, Visualization

**Islem Mejri:** Validation, Visualization, Writing – Review & Editing

**Tasnim znegui:** Visualization, Writing – Review & Editing

**Wafa Fehri**: Supervision, Validation, Visualization, Writing – Review & Editing

## Data availability

### Underlying data

Figshare: ECHOCARDIOGRAPHIC EVALUATION IN CHILDREN WITH SICKLE CELL ANEMIA: CONTRIBUTION OF 2D STRAIN.xlsx. https://doi.org/10.6084/m9.figshare.20949031.v4. ^31^

Data are available under the terms of the Creative Commons Attribution 4.0 International license (CC-BY 4.0).
