# Supplementary material for: Evaluation of left ventricular systolic function in children with sickle cell anemia: contribution of 2D strain
Source: F1000Res. 2022 Dec 5;11:1207. Originally published 2022 Oct 24. [Version 2] doi: 10.12688/f1000research.125345.2 (PMC9732499; doi:10.12688/f1000research.125345.2)
Supplement: Peer review history

# Peer review: Reviewer response for version 2

**DOI:** 10.5256/f1000research.141488.r157300

**Published:** 2022-12-08

**Authors:** Anthony Matta

**Affiliations:** 1 Heart Failure Unit, Department of Cardiology, Institute CARDIOMET, University Hospital of Toulouse, Toulouse, France

The authors have addressed the raised points in an appropriate way. The manuscript is well improved. Then, the current version of paper is suitable for indexing.

Is the work clearly and accurately presented and does it cite the current literature?

No

If applicable, is the statistical analysis and its interpretation appropriate?

Yes

Are all the source data underlying the results available to ensure full reproducibility?

Partly

Is the study design appropriate and is the work technically sound?

Partly

Are the conclusions drawn adequately supported by the results?

Partly

Are sufficient details of methods and analysis provided to allow replication by others?

Partly

Reviewer Expertise:

Cardiovascular disease

I confirm that I have read this submission and believe that I have an appropriate level of expertise to confirm that it is of an acceptable scientific standard.

# Peer review: Reviewer response for version 1

**DOI:** 10.5256/f1000research.137642.r154030

**Published:** 2022-11-03

**Authors:** Anthony Matta

**Affiliations:** 1 Heart Failure Unit, Department of Cardiology, Institute CARDIOMET, University Hospital of Toulouse, Toulouse, France

I have reviewed the submitted paper entitled “Evaluation of left ventricular systolic function in children with sickle cell anemia: contribution of 2D strain”. The authors showed that left ventricular global longitudinal strain is significantly altered in the SCD-group. They also suggested that treatment with hydroxycarbamide is a predictive factor.

 This paper is not well written and not suitable for indexing in the current form. Major revision is required before being considered for further evaluation. Herein, I addressed the main points:

1. English editing is needed through all the manuscript.
2. The authors must define each abbreviation before its first use in the text (C means control in the abstract section).
3. Conclusion in abstract section is out of nowhere. 
4. In the introduction section, the authors should cite the potential cardiac complications of sickle cell disease and explain in detail the two-dimensional strain technique.
5. Methods section. It is completely unclear how the authors have performed this study. First, it is a case control study. Inclusion and exclusion criteria are not well defined. They mentioned among inclusion criteria: “having SCA and a performed TTE”, then they mentioned in the text that they performed TTE for all patients. So why do they fix the performance of TTE during follow-up as an inclusion criterion? When have they performed TTE? Did they compare the performed TTE to the previous ones?
6. What was the indication for TTE in SCA or controls group? What was the indication for hospital admission in controls group?
7. A comparative table with 4 columns should be added in the result section (Variable, SCA-group, C-group, p-value). This table should figure baseline characteristics (age, sex, BMI, laboratory values (Hb), treatment, dose, mean of LVGLS, LVEF, etc.).
8. How much was the proportion of patients with impaired LVGLS? Also, a clear definition of impaired LVGLS is lacking.
9. Multivariable logistic regression investigating the association between impaired LVGLS and other parameters (Hydroxycarbamide dose, osteonecrosis, splenomegaly) was not performed. Then, a conclusion only based on bivariate analysis is extremely weak.
10. I suggest putting 3 main tables in the results section: Table 1 (point 6), Table 2 (Impaired LVGLS vs normal LVGLS), Table 3 (multivariable logistic regression).
11. Was a single TTE performed to each study participant? Or were repeated TTE performed?
12. Clear definitions of left ventricular dilation and hypertrophy were missed. All these terms must be defined in the method section including for threshold of normal LVGLS
13. The authors should mention in the result section that LVGLS was normal in all study participants in C-group.
14. It is true that there was a significant difference in echocardiographic parameters between study groups, but the represented values are within range of normal in both groups. They were not suitable for neither hypertrophic nor dilated cardiomyopathy diagnosis.
15. The authors should represent their results in the first paragraph of the discussion section. Then, they must focus on discussing their findings based on literature data.
16. They claim that hydroxycarbamide treatment is the predictor of LVGLS impairment, but in fact, the hydroxycarbamide dosage differs significantly between study groups. In the concerned table, the authors differ hydroxycarbamide treatment from hydroxycarbamide dose.
17. The authors should explain what this study adds to what is already known in the field. Previous studies investigate the role of LVGLS to predict cardiac outcomes in SCD patients.
18. Re-write the conclusion (rephrase the first sentence which must highlight your take-home message).
19. It is unclear if a LVGLS below -20% is a predictor for cardiac disease in the future. It could be an incidental finding without clinical relevance. Usually, A GLS higher than 18% is considered normal. 

Is the work clearly and accurately presented and does it cite the current literature?

No

If applicable, is the statistical analysis and its interpretation appropriate?

Yes

Are all the source data underlying the results available to ensure full reproducibility?

Partly

Is the study design appropriate and is the work technically sound?

Partly

Are the conclusions drawn adequately supported by the results?

Partly

Are sufficient details of methods and analysis provided to allow replication by others?

Partly

Reviewer Expertise:

Cardiovascular disease

I confirm that I have read this submission and believe that I have an appropriate level of expertise to state that I do not consider it to be of an acceptable scientific standard, for reasons outlined above.

# Response

**Published:** 2022-11-22

**Authors:** Tasnim Znegui

**Affiliations:** Tunis El Manar University, Tunisia

**1. English editing is needed through all the manuscript.**

 We revised the manuscript.

**2. The authors must define each abbreviation before its first use in the text (C means control in the abstract section).**

 We rectified all abbreviations in the text.

**3. Conclusion in abstract section is out of nowhere.**

 We corrected the conclusion in the abstract section.

**4. In the introduction section, the authors should cite the potential cardiac complications of sickle cell disease and explain in detail the two-dimensional strain technique.**

 We added a paragraph to explain this.

**5. Methods section. It is completely unclear how the authors have performed this study. First, it is a case control study. Inclusion and exclusion criteria are not well defined. They mentioned among inclusion criteria: “having SCA and a performed TTE”, then they mentioned in the text that they performed TTE for all patients. So why do they fix the performance of TTE during follow-up as an inclusion criterion? When have they performed TTE? Did they compare the performed TTE to the previous ones?**

 It’s a cross sectional case-control study

 The (SCA) group included children followed up at the pediatric department of the Tunis military hospital for sickle cell disease during the study period. We did not include patients with SCA with history of heart diseases, or those who had undergone cardiac surgery.

 The control (C) group selected 30 children with no history of cardiovascular or respiratory pathology and not presenting anemia at the time of the study.  Then, C group was matched to the SCA group by age, sex and body mass index.

**6. What was the indication for TTE in SCA or controls group? What was the indication for hospital admission in controls group?**

 These children had been hospitalized in the pediatric department and referred to a pediatric cardiology consultation for assessment of a heart murmur or for exploration of atypical chest pain, and who had a normal echocardiography.

**7. A comparative table with 4 columns should be added in the result section (Variable, SCA-group, C-group, p-value). This table should figure baseline characteristics (age, sex, BMI, laboratory values (Hb), treatment, dose, mean of LVGLS, LVEF, etc.).**

 We added a table untitled ‘Comparison of baseline characteristics of the two groups’.

**8. How much was the proportion of patients with impaired LVGLS? Also, a clear definition of impaired LVGLS is lacking.**

 In SCA group, LVGLS was estimated at -21.2±3%. It was significantly decreased (< -20%) in 46% of children (n=14) referring to ‘Reference Ranges of Left Ventricular Strain Measures by Two-Dimensional Speckle-Tracking Echocardiography in Children A Systematic Review and Meta-Analysis. J Am Soc Echocardiogr. 2016 Mar;29(3):209-225.e6. Epub 2015 Dec 30.

**9. Multivariable logistic regression investigating the association between impaired LVGLS and other parameters (Hydroxycarbamide dose, osteonecrosis, splenomegaly) was not performed. Then, a conclusion only based on bivariate analysis is extremely weak.**

 In multivariate analysis we found a correlation between LVM and impaired GLS (b = - 0.082, p<0.001)

**10. I suggest putting 3 main tables in the results section: Table 1 (point 6), Table 2 (Impaired LVGLS vs normal LVGLS), Table 3 (multivariable logistic regression).**

 We have organized the results section.

**11. was a single TTE performed to each study participant? Or were repeated TTE performed?**

 A single TTE was performed to each study participant.

**12. Clear definitions of left ventricular dilation and hypertrophy were missed. All these terms must be defined in the method section including for threshold of normal LVGLS**

 We revised the methodology.

**13. The authors should mention in the result section that LVGLS was normal in all study participants in C-group.**

 We mentioned this result.

**14. It is true that there was a significant difference in echocardiographic parameters between study groups, but the represented values are within range of normal in both groups. They were not suitable for neither hypertrophic nor dilated cardiomyopathy diagnosis.**

 We added a Table to compare the LV morphological parameters and LV systolic function in the two groups.

**15. the authors should represent their results in the first paragraph of the discussion section. Then, they must focus on discussing their findings based on literature data.**

 We corrected the first paragraph of the discussion section.

**16. they claim that hydroxycarbamide treatment is the predictor of LVGLS impairment, but in fact, the hydroxycarbamide dosage differs significantly between study groups. In the concerned table, the authors differ hydroxycarbamide treatment from hydroxycarbamide dose.**

 We corrected the correlation between treatment and LVGLS impairment.

**17. the authors should explain what this study adds to what is already known in the field. Previous studies investigate the role of LVGLS to predict cardiac outcomes in SCD patients.**

 The aim of this study was to assess the contribution of 2D strain to the detection of subclinical LV myocardial damage in children with SCA and to reveal associated factors *with abnormal LVGLS.*

**18. Re-write the conclusion (rephrase the first sentence which must highlight your take-home message).**

 We have corrected the conclusion.

# Response

**Published:** 2022-11-23

**Authors:** Tasnim Znegui

**Affiliations:** Tunis El Manar University, Tunisia

We are grateful to the reviewers for their insightful comments.

# Peer review: Reviewer response for version 1

**DOI:** 10.5256/f1000research.137642.r154031

**Published:** 2022-10-31

**Authors:** Hassen Ibn Hadj Amor

**Affiliations:** 1 Cardiology Department, Tahar Sfar Hospital, Mahdia, Tunisia; 2 University of Monastir, Monastir, Tunisia

Sickle cell disease is endemic in north Africa. It used to be a life-threatening disease with reduced life expectancy which improved in the recent years due to healthier lifestyles, medical monitoring, and new therapies. Nevertheless, we attest to an increase of chronic multi-systemic complications in addition to acute complications, mainly cardiovascular ones. The authors highlighted in this paper the importance of early detection of myocardial damage using echocardiographic techniques such as two-dimensional (2D) strain during childhood. It is a patient control study with a predictive factor analysis for alteration of the global longitudinal strain of the left ventricle. Generally, the manuscript is well written and the topic is quite interesting for the reader. The paper seems suitable for indexing in its actual version.

Is the work clearly and accurately presented and does it cite the current literature?

Yes

If applicable, is the statistical analysis and its interpretation appropriate?

Yes

Are all the source data underlying the results available to ensure full reproducibility?

Yes

Is the study design appropriate and is the work technically sound?

Yes

Are the conclusions drawn adequately supported by the results?

Yes

Are sufficient details of methods and analysis provided to allow replication by others?

Yes

Reviewer Expertise:

cardiology, echocardiography

I confirm that I have read this submission and believe that I have an appropriate level of expertise to confirm that it is of an acceptable scientific standard.
